# A clinically managed weight loss program evaluation and the impact of COVID-19

**DOI:** 10.3389/fnut.2023.1167813

**Published:** 2023-06-16

**Authors:** Katrina Cachero, Rebecca Mollard, Semone Myrie, Dylan MacKay

**Affiliations:** ^1^Department of Food and Human Nutritional Sciences, University of Manitoba and the Chronic Disease Innovation Centre, Winnipeg, MB, Canada; ^2^Chronic Disease Innovation Centre, Seven Oaks General Hospital, Winnipeg, MB, Canada

**Keywords:** weight loss, weight loss program, obesity, clinically managed weight loss program, evaluation

## Abstract

**Introduction:**

With the prevalence of obesity increasing, many weight-loss programs were created to aid in combating the trend. The Weight Loss Clinic (WLC) was created to provide personalized support for lifestyle changes using a multidisciplinary team with medical oversight. This study evaluated the clinically-managed weight loss program at the Wellness Institute.

**Methods:**

This was a prospective evaluation of a newly established program between January 2019–August 2020. Participants who entered the weight loss program were approached to learn about the evaluation. A total of 41 participants were included. The primary outcomes included changes in body weight and achievement of more than 5% initial body weight loss. Outcome measures were collected pre-and post-program and the data was analyzed through paired t-tests on R studio.

**Results:**

Greater body weight-loss was seen in completers pre-COVID-19 compared to those who completed during the pandemic (Mean, ±SD; 7.51 ± 6.24 kg *n* = 13 *p* < 0.001 vs. 1.75 ± 4.43 kg *n* = 9, *p* = 0.02). Completers pre-COVID-19 demonstrated improvements in waist circumference, Framingham risk score, blood pressure, hemoglobin A1C, and body fat percentage.

**Conclusions and implications:**

Though the sample size was small to show definitve evidence, the results may suggest the program worked well prior to the pandemic but the pandemic created barriers to weight-loss for participants.

## 1. Introduction

Almost two-thirds of Canadian adults are living with overweight or obesity (1), which can increase the stress on the health system via increased risk for chronic diseases (2) including type-2 diabetes and cardiovascular diseases. Weight reduction is well documented to improve cardiovascular disease risk factors such as the lowering of high blood pressure, low density lipoprotein (LDL)-cholesterol, and triglycerides (3). Weight reduction is also associated with reduced all-cause mortality in individuals living with overweight and obesity (4).

While the market is overwhelmed by weight loss products and services that are often non-evidence based, there is a gap in evidence-based, professionally delivered clinical weight loss services in Canada. The 2020 Canadian Clinical Practice Guidelines (CCPG) for obesity management recommends that interventions for adults living with obesity should follow individualized care plans that address the root causes of obesity and provide support for behavioral change such as diet and lifestyle intervention, psychological intervention, pharmacotherapy, or bariatric surgery (2, 5). As summarized in the CCPG, living with obesity is multi-factorial and involves a variety of factors, including not only weight, but sleep, quality of life and mental health. The Wellness Institute (WI) is a self-supporting non-profit organization that operates as a medical fitness facility attached to the Seven Oaks General Hospital (SOGH) in Winnipeg, Manitoba. A 2015 outcome analysis conducted with WI members indicated that 51% of new members had high blood sugars and blood pressure, and/or were living with overweight or obesity (6, 7). In response to these identified risks, the WI developed a clinically managed weight loss program focused not only on body weight reduction but also improvement in cardiometabolic risk factors. This new program, known as the Weight Loss Clinic (WLC) provides personalized support for individuals to ensure the program is customized to participants’ needs. The program is managed by a clinical team including a program manager, registered dietitians (RD), Canadian Society of Exercise Physiology (CSEP)-certified personal trainers (CCPTs), a clinical psychology associate (CPA) or a cognitive behavioral therapist (CBT), and a physician. With the focus on improvement in cardiometabolic risk factors, the program assesses body mass index (BMI), lipid profile, and Framingham risk scores (8). To further guide assessments by the team, general self-efficacy (9) and components of nutrition are measured, such as mindful eating, cognitive restraint, and emotional eating (10, 11).

In March 2020, Coronavirus disease 2019 (COVID-19) caused by severe acute respiratory syndrome coronavirus 2 (SARS-CoV-2) was declared a global pandemic, changing the lives of everyone around the globe. A number of public health measures were implemented to combat the spread of COVID-19 with varying success (12). These include lockdowns and quarantine orders which have resulted in increases in both unhealthful dietary behavior and sedentary behavior, and declines in mental health (13). Thus, COVID-19 and resulting public health orders may have increased obesogenic behaviors and the potential for weight gain (14). In Manitoba, the pandemic resulted in restrictions which impacted the delivery of the WLC. This study describes an evaluation of participating in the WLC, which was ongoing when the COVID-19 pandemic began, creating a natural experiment of the impact of a global pandemic on a clinically managed weight loss program.

## 2. Materials and methods

### 2.1. Participants and setting

This study was a prospective evaluation of a newly established weight loss program between January 2019 and August 2020. The inclusion criteria of the program were individuals over the age of 18 years old and living with overweight or obesity (BMI > 25 kg/m^2^), who had been told by a physician or primary care provider to lose weight, and had tried to lose weight in the past but had been unsuccessful at maintaining weight loss. Women who were pregnant or lactating were not eligible for the program. All potential participants who entered the program were approached and asked if they would like to participate in a program evaluation. Participants gave informed consent before participating in the evaluation. The WLC is located in the WI at SOGH in Winnipeg, Manitoba, Canada. The data was collected at the Chronic Disease Innovation Centre (CDIC), a non-profit health research institute located at SOGH that provides independent research services for academic, industry, and non-profit partners including WI.

This study was conducted in accordance with the Declaration of Helsinki, and received approval from the Manitoba Research Ethics Board [Ethics # HS22267 (H2018:401)]. It was registered on Clinicaltrials.gov (ID# NCT04290910) retrospectively as it was a program evaluation and not a clinical trial. Informed consent was obtained from all participants involved in the study.

### 2.2. Program design

The WLC was delivered in-person by a team, including a program manager, physician, RD, CCPT and CPA/CBT. The team collaborated to prescribe a plan best suited for the participants’ needs. Appointments with the RD and CCPT consisted of seven 1-h appointments and seven 30-min appointments each (details shown in Supplementary Figure 1). The weight loss program was based on sustaining behavioral changes and was also tailored to improve lifestyle, sleep, and mental health. During the initial and exit assessments, the CCPT collected self-reported health conditions, medications and cardiovascular biomarkers such as, BMI, blood pressure, lipid profile, and Framingham risk score. The Framingham Risk Score was categorized into low, intermediate or high and was used to estimate a 10-year cardiovascular disease risk (8). An oscillometric blood pressure monitor was used to collect blood pressure and heart rate. Non-fasted blood samples were collected from participants by a trained phlebotomist and analyzed for lipid profile and hemoglobin A1C at the Diagnostic Services of Manitoba in Winnipeg, Canada (15).

The program evolves in a 3-stage progression, which is explained in detail in Supplementary Figure 1. The first stage (Day 1–7) is the assessment phase, this is where participants share what they are looking to achieve and assessing their stage of readiness to change. Each discipline also has the opportunity to assess the participants based on their area of expertise. The second stage is the intensive phase (Day 18–21), this is where the participants receive a nutrition prescription based on the RD’s assessment, this may include a meal plan from EatLove website/app or nutritional goals (6). The RD used information gathered from the three-factor questionnaire (11), mindful eating questionnaire (10), and 3-day food recall (these were filled out at the initial assessment) and a one-on-one appointment to further guide the intervention. In terms of exercise, the CCPT used information from the initial assessment questionnaire to guide and provide a personalized exercise prescription through the Wellness App (6). A one-hour assessment is included during the initial phase. A psychological intervention may have replaced the nutrition intervention at this stage if the participant screened for severe depression, anxiety, or disordered eating, or if they presented with any psychosocial concerns. The psychological intervention includes support for overcoming challenges and barriers, help understanding stages of change, and cognitive behavioral therapy. The third stage, the transformation phase (Day 22–119), was the remaining 105 days in the program and includes sessions with both the RD and CCPT. The nutrition and exercise prescriptions were modified depending on lifestyle changes and/or progress that the participant made. The participants had a total of 12 h each with the RD and CCPT. After 119 days in the program, the participants were given the options to continue in the transformation phase, enter the maintenance phase, or exit the program. During the maintenance phase, participants had two meetings per month with the RD and CCPT. This phase is available to help routinize the habits participants learned in previous phases. The maintenance phase was not included in this evaluation.

The program was delivered in-person and in-center for those who completed prior to March 2020. With the impact of COVID-19, the program was delivered virtually for those who joined the program after March 2020. Given that the program was still running during COVID-19, there were a few participants that started in-person and ended virtually.

### 2.3. Program study design

There were no changes to the program evaluation pre-and during COVID. Program success was defined as achieving weight loss of more than 5% of initial body weight. Due to the personalized nature of the program, the number of days the participant attended and how much weight was lost was used to further guide the success of the program. To further evaluate program effectiveness, baseline and exit measures were collected, which included cardiovascular disease risk factors, clinical chemistry, and dietary behavior. They were then compared to reference values to indicate whether there was improvement in measurements. Participants who completed the program were asked to fill out an exit survey assessing acceptability of the program, participant feedback, and program experience. The exit survey was created by the WLC team and is provided under Supplementary material.

### 2.4. Outcome measures

The primary outcomes included changes in body weight and achievement of more than 5% initial body weight loss. The secondary outcomes include changes in BMI, waist circumference, blood pressure, heart rate, Framingham risk score, blood lipids, hemoglobin A1C, body fat percentage, sleep quality (16), quality of life (17), general self-efficacy (9), mindful eating (10), cognitive restraint (11), and emotional eating (11). These were assessed through questionnaires, including the Pittsburgh sleep quality index, SF-36 quality of life, general self-efficacy scale, three-factor eating questionnaire, and mindful eating questionnaire. Measurements were taken prior to starting the program and after completion of the transformation phase. For those who did not complete the program, all measures up until the participant exited were collected.

### 2.5. Data analysis

Statistical analysis was performed using R Studio (R Studio, Boston, MA, United States) (18). The effects of participation in the weight loss program on the captured outcomes were analyzed by the R generalized linear model function using a pre-post design. To evaluate the impact of COVID-19, the completed groups were separated into pre-and during COVID-19. We included age and sex as fixed factors and compared the last measurement taken for each outcome measure to baseline, pairing by participant. For all analyses, value of ps less than 0.05 were considered significant. Descriptive statistics, such as attendance and participant feedback were also measured and reported. Results were reported as mean +/− standard deviation and frequency and percentage for continuous and categorical variables, respectively.

## 3. Results

A total of 43 participants were enrolled in the evaluation; 2 decided not to continue, 16 did not complete, 25 completed and 22 were included at the time of analysis (the remaining three participants completed the program but were not included at the time of analysis due to no exit data collected, the participants declined to come into the facility and have their exit data collected due to concerns around COVID-19). For those that did not complete the program, reasons for incompletion pre-COVID-19 included loss to follow up (*n* = 4), medical reasons unlinked to the program (*n* = 3), family reason (*n* = 1), program hold (*n* = 1), decided not to continue (*n* = 1), not a good fit right now (*n* = 1), and moved out of the country (*n* = 1), whereas, for those that completed during COVID-19, reasons for incompletion included loss to follow up (*n* = 2), decided not to continue (*n* = 1) and encountered medical reasons requiring stoppage unlinked to the program (*n* = 1).

Participants were 46 ± 12.52 years old with majority female (Table 1). At baseline, cardiovascular disease risk markers, such as blood pressure, Framingham risk score, and lipid profile were within normal range for most participants, both male and female. As expected, weight, BMI and body fat percentage were higher than the ideal range (Table 1). There were some participants who declined to be measured or provide measurements for certain outcomes, hence the variations in sample size found in Tables 2, 3. Overall, participants who completed the program lost 5.15 ± 5.18 kg, *p* < 0.001, *n* = 22 (Table 2). Since we were unable to collect exit measurements on participants who did not complete, their last measurement taken was collected. Participants who did not complete remained in the program for an average of 80 ± 47.23 days and lost−0.91 ± 2.71 kg, *p* = 0.10, *n* = 9. There were three participants who joined the program pre-COVID-19 and completed during COVID-19.

**Table 1:** Participant characteristics at baseline.

Parameter	*N*	Baseline mean ± SD					Ideal range for parameters
		Total	*n*	Female	*n*	Male	
Age (years)	43	46.76 ± 12.52	38	46.34 ± 12.95	5	50.00 ± 9.08	
Weight (kg)	41	114.25 ± 30.44	36	108.43 ± 26.72	5	156.16 ± 22.9	
Total BMI (kg/m^2^)	41	40.72 ± 9.30	36	37.02 ± 9.01	5	47.08 ± 5.69	Underweight < 18.5
							Healthy Weight 18.5–24.9, Overweight 25–29.9
							Obesity Class I 30–34.9
							Obesity Class II 35–39.9
							Obesity Class III > 40
BMI overweight (kg/m^2^)	4	28.47 + 0.65	4	28.47 + 0.65	0	-	25–29.9
BMI Obesity class I (kg/m^2^)	10	32.87 ± 1.14	10	32.87 ± 1.14	0	-	30–34.9
BMI Obesity class II (kg/m^2^)	8	37.35 ± 1.45	8	37.35 ± 1.45	0	-	35–39.9
BMI Obesity class III (kg/m^2^)	19	48.78 ± 7.07	14	49.39 ± 7.60	5	47.08 ± 5.69	Obesity Class III > 40
Waist circumference (cm)	40	124.27 ± 21.23	35	119.02 ± 17.29	5	149.94 ± 14.04	<88 cm (F) (19)
							<102 cm (M) (19)
Systolic blood pressure (mmHg)	41	127.67 ± 15.08	36	125.37 ± 13.27	5	131.60 ± 11.06	<130 mmHg (20)
Diastolic blood pressure (mmHg)	41	81.78 ± 7.67	36	80.17 ± 6.71	5	86 ± 8.57	<85 mmHg (20)
Heart rate (beats per minute)	41	75.18 ± 11.12	36	73.11 ± 10.33	5	77.54 ± 9.48	60–80 beats per min (21)
Total blood cholesterol (TC) (mmol/L)	41	4.90 ± 1.12	36	5.06 ± 1.03	5	3.55 ± 0.47	<5.2 mmol/L (22)
HDL cholesterol (mmol/L)	41	1.30 ± 0.38	36	1.36 ± 0.40	5	3.55 ± 0.47	>1.3 mmol/L (F) (22)
							>1.0 mmol/L (M) (22)
LDL cholesterol (mmol/L)	41	2.76 ± 0.86	36	2.86 ± 0.80	5	1.89 ± 0.47	<3.5 mmol/L (22)
TC/HDL cholesterol ratio	40	3.89 ± 1.27	35	3.94 ± 1.36	5	3.48 ± 0.67	<4 (F) (23)
							<5.2 (M) (23)
Triglycerides (mmol/L)	41	1.85 ± 0.91	36	1.85 ± 0.99	5	1.50 ± 0.44	≤1.7 mmol/L (22)
Hemoglobin A1C (%)	40	5.78 ± 0.67	35	5.74 ± 0.53	5	5.72 ± 0.72	4%–6% (24)
Body fat percentage (%)	41	49.40 ± 5.21	36	49.21 ± 5.32	5	46.72 ± 3.63	18%–28% (F) (25)
							10%–20% (M) (25)
Skeletal muscle mass (kg)	41	31.90 ± 8.38	36	30.00 ± 5.50	5	47.0 ± 5.56	
Body fat mass (kg)	40	56.35 ± 18.21	35	52.06 ± 16.39	5	73.39 ± 15.81	
Total Framingham risk score (%)	35	6.85 ± 5.46	30	6.28 ± 5.46	5	10.26 ± 4.53	Low < 10%
							Intermediate 10%–19%
							High ≥ 20%
Low Framingham risk score (%)	30	5.17 ± 3.01	28	5.14 ± 3.11	2	5.6 ± 0.0	<10%
Intermediate Framingham risk score (%)	4	14.33 ± 2.63	1	17.2 ± 0.0	3	13.37 ± 2.20	10%–19%
High Framingham risk score (%)	1	27.4 ± 0.0	1	27.4 ± 0.0	0	-	>20%

**Table 2:** The mean differences (baseline and exit) of weight, BMI and percent body weight loss for the non-completers, completers, completers pre-COVID-19 and completers during COVID-19.

Change in Parameters	Non-completers (*n* = 9)		All completers (*n* = 22)		Completers pre-COVID-19 (*n* = 13)		Completers during COVID-19 (*n* = 9)		*p*-value for completers pre-COVID-19 vs. completers during COVID-19
	Mean difference ± SD	*p*-value	Mean difference ± SD	*p*-value	Mean difference ± SD	*p*-value	Mean difference ± SD	*p*-value	
Weight (kg)	-0.91 ± 2.71	0.1063	−5.15 ± 5.18	0.0008	−7.51 ± 6.24	0.0009	−2.05 ± 4.39	0.1985	0.025
BMI (kg/m^2^)	−0.13 ± 0.90	0.2981	−1.97 ± 2.33	0.0007	−2.68 ± 2.00	0.0004	−0.18 ± 1.26	0.495	0.002
Percent body weight loss (%)	−1.50 ± 1.95	0.0734	−4.78 ± 4.85	0.0005	−6.34 ± 4.25	0.0009	−2.52 ± 5.01	0.1985	0.0807

**Table 3:** The mean differences (baseline and exit) for the participants who have completed the program, grouped by those who completed pre-COVID-19 and during COVID-19.

Change in Parameters	Completers pre-COVID-19 *n* = 13			Completers during COVID-19 *n* = 9			*p*-value for completers pre-COVID-19 vs. completers during COVID-19
	*n*	Mean difference ± SD	*p*-value	*n*	Mean difference ± SD	*p*-value	
Waist circumference (cm)	13	−6.54 ± 4.50	0.0002	9	−5.04 ± 7.31	0.072	0.5936
Framingham risk score (%)	12	−2.43 ± 2.61	0.008	7	−2.04 ± 3.11	0.1327	0.7848
Systolic blood pressure (mmHg)	13	−9.33 ± 9.87	0.005	9	−5.52 ± 8.68	0.093	0.3512
Diastolic blood pressure (mmHg)	13	−5.51 ± 5.93	0.006	9	−4.39 ± 6.30	0.070	0.6804
TC (mmol/L)	12	−0.04 ± 0.61	0.828	9	−0.02 ± 0.50	0.903	0.9416
HDL cholesterol (mmol/L)	12	0.034 ± 0.09	0.226	8	−0.04 ± 0.21	0.542	0.3201
LDL cholesterol (mmol/L)	12	−0.098 ± 0.35	0.353	8	0.12 ± 0.31	0.281	0.1497
Triglycerides (mmol/L)	12	0.004 ± 0.60	0.981	8	−0.20 ± 0.49	0.243	0.3894
Hemoglobin A1C (%)	12	−0.13 ± 0.20	0.04	8	−0.1 ± 0.28	0.3198	0.768
Body fat percentage (%)	13	−2.72 ± 1.81	0.0001	9	−0.99 ± 2.56	0.2801	0.1033
Skeletal muscle mass (lbs)	13	−1.51 ± 3.23	0.117	9	−4.9 ± 13.46	0.3068	0.4793
Body fat mass (kg)	13	−6.59 ± 4.73	0.0002	7	−1.56 ± 4.27	0.3696	0.0295

Participants who completed pre-COVID 19 lost 7.51 kg ± 6.24 kg, *p* < 0.001, *n* = 13), this is an average of 6.34% (*p* < 0.001) initial body weight loss. There were improvements in waist circumference (−6.54 ± 4.50 cm, *p* < 0.001, *n* = 13), Framingham risk score (−2.43 ± 2.61%, *p* = 0.008, *n* = 12), systolic blood pressure (−9.33 ± 9.87 mmHg, *p* = 0.005, *n* = 13), diastolic blood pressure (−5.51 ± 5.93 mmHg, *p* = 0.006, *n* = 13), hemoglobin A1C (−0.13 ± 0.20%, *p* = 0.04, *n* = 12, body fat percentage (−2.72 ± 1.81%, *p* < 0.001, *n* = 13), and body fat mass (−6.59 ± 4.73 kg, *p* < 0.001, *n* = 13) in completers pre-COVID-19 (Table 3) compared to baseline. For participants who completed pre-COVID-19, we saw an improvement in cognitive restraint (+4.62 ± 4.88, *p* = 0.007, *n* = 12), decline in uncontrollable eating (−4.42 ± 4.35, *p* = 0.005, *n* = 12) and emotional eating (−1.37 ± 1.23, *p* = 0.002, *n* = 12), and improvement in general health (+18.33 ± 17.32, *p* = 0.013, *n* = 9) based on the questionnaires (Table 4). There were no improvements in the other SF-36 components, such as, physical functioning, emotional problems, energy/fatigue, emotional well-being, social functioning and pain. There were improvements in sleep (−1.09 + 2.84, *p* = 0.23, *n* = 11) but are considered statistically insignificant.

**Table 4:** The mean differences (baseline and exit) for questionnaire data on the participants who have completed the program, grouped by those who completed pre-COVID-19 and during COVID-19.

Change in Questionnaires	Completers pre-COVID-19			Completers during COVID-19			*p*-value
	*n*	Mean difference ± SD	*p*-value	*n*	Mean difference ± SD	*p*-value	
General self-efficacy scale	12	−0.79 ± 4.90	0.5873	7	0.86 ± 2.19	0.341	0.3297
Mindful eating	12	−0.26 ± 0.23	0.0024	7	−0.54 ± 0.79	0.1176	0.3833
Three factor eating—cognitive restraint	12	4.62 ± 4.88	0.0073	7	4.07 ± 4.15	0.041	0.7965
Three factor eating—uncontrollable eating	12	−4.42 ± 4.35	0.005	7	−3.14 ± 3.34	0.047	0.485
Three factor eating—emotional eating	12	−1.37 ± 1.23	0.0025	7	0.143 ± 3.62	0.9204	0.32
Pittsburgh sleep quality index score	11	−1.09 ± 2.84	0.2322				0.3894
SF-36 physical functioning	9	8.33 ± 11.45	0.060				0.4594
SF-36 role limitations due to physical health	9	30.55 ± 41.03	0.055				0.8853
SF-36 role limitations due to emotional problems	9	14.82 ± 42.44	0.3463				0.2534
SF-36 energy/fatigue	9	10.55 ± 22.56	0.1981				0.9072
SF-36 emotional well-being	9	6.67 ± 11.49	0.1199				0.0372
SF-36 social functioning	9	−5.83 ± 10.40	0.131				0.0947
SF-36 pain	9	−0.05 ± 23.65	0.9945				0.1927
SF-36 general health	9	18.33 ± 17.32	0.013				0.4204

Participants who completed during COVID-19 lost (2.05 kg ± 4.39, *p* = 0.19, *n* = 9), this is an average of 1.5% (*p* = 0.07) initial body weight loss. The remaining outcomes (waist circumference, Framingham risk score, systolic blood pressure, diastolic blood pressure, hemoglobin A1C, body fat percentage, body fat mass) remained unchanged compared to baseline. For participants who completed during COVID-19, quality of life, sleep and nutrition outcomes remained unchanged compared to baseline.

The changes in body weight in participants in all completers pre-COVID, during COVID and non-completers are plotted in Supplementary Figure 3. It shows that completers pre-COVID lost more weight than completers during COVID-19, where completers during COVID-19 may have actually gained weight.

For non-completers, the last body weight attained by the program was used. Completers pre-COVID-19 lost more than 5% of their initial body weight, whereas, only 11% of completers during COVID-19 achieved the same. The impact of COVID-19 on weight loss is plotted in Supplementary Figure 4. Participants who completed pre-COVID-19 lost more body weight than completers during COVID-19. The impact of program duration on weight loss as a percentage is shown in Supplementary Figure 5. Although it was available, the nutritional intervention was not replaced by psychological intervention for any of the participants in the evaluation.

### 3.1. Exit survey results

The results of our exit survey showed that 73% (16 out of 22) of participants used the Wellness App, 86% (19 out of 22) of participants used the EatLove software for meal planning and preparation, 72% (17 out of 22) of participants gained knowledge, skills, and confidence as the program went on, and 82% (18 out of 22) of participants reported making valuable lifestyle changes. In terms of the satisfaction of the different components of the program, 85% (17 out of 20) reported satisfaction with the nutrition portion, 100% (21 out of 21) reported satisfaction with the exercise portion, and 50% (10 out of 20) reported satisfaction with the psychology portion. As well, 100% (22 out of 22) of participants reported they would recommend the weight loss program to a friend or family member.

## 4. Discussion

The evaluation results suggest that participating in the program in-person resulted in health benefits, reduced adiposity and improved cardiometabolic health, however, some outcomes were not seen in some participants due to barriers created by the COVID-19 pandemic. When COVID-19 was declared a pandemic in March 2020, life around the world changed. In Manitoba, the pandemic lockdowns and restrictions made in-person delivery of the weight loss program more difficult or even impossible in some cases. Thus the delivery of the program was done virtually or through telephone with the same number of hours pre-COVID-19 allocated per WLC team member. The program was designed to be delivered in-person, not virtually, and for a virtual program to be successful there may be a need for additional support including online digital platforms.

For a four-month weight loss program, the ideal weight lost is between 7.3 to 14.5 kg (one to two pounds per week) (2, 4). The average weight lost was 7.51 kg for those who completed the program pre-COVID-19, which is clinically significant for a 4-month weight loss program. In comparison, minimal changes in body weight were observed for those that completed during COVID-19. The changes from the COVID-19 pandemic may have had a negative impact on the program. As shown on Supplementary Figures 3, 4, it showed that participants who took part pre-COVID-19 lost more weight than those who took part during COVID-19. This could be due to the conditions created by the COVID-19 pandemic including lockdowns and quarantines. A study by Zachary et al. identified risk factors for weight gain during self-quarantine including a lack of dietary restraint, eating in response to stress, and reduced physical activity (16). Additionally, many people have reported weight gain during the pandemic (16, 26, 27), Marchitelli et al. (28) reported weight gain during lockdown in participants with and without a psychiatric diagnosis (16, 26). Lockdown and quarantine orders have been shown to change dietary behaviors, increase sedentary behaviors, and worsen mental health, with individuals living with obesity showing greater increases to unhealthful dietary behaviors and declines in mental health (15). It is possible that participating in the weight loss program during COVID-19 may have helped in weight maintenance, given that the participants were at a high risk for weight gain. Participants who completed pre-COVID-19 showed improvements in dietary behaviors such as, mindful eating, cognitive restraint, uncontrollable eating, and emo-tional eating, whereas there were no changes shown in these measures for those who completed during COVID-19. An evaluation by the Look AHEAD Research Group (29) showed that weight loss achieved with health behavioral changes is usually 3%–5% of body weight, which can result in meaningful improvements in obesity-related comorbidities. Those that com-pleted the program pre-COVID-19 lost an average of 6.34% body weight.

There were also improvements in the secondary outcomes for those that completed the program pre-COVID-19, including waist circumference, systolic blood pressure, diastolic blood pressure, and body fat percentage. These improvements would suggest a decreased risk of obesity-related morbidities, such as type 2 diabetes (30), and hypertension (31). Conversely, those that participated during-COVID-19 saw no changes in these measures. However, it is important to note that average blood pressure, cholesterol and blood glucose values at baseline were within the ideal range, suggesting that the population in the study was not experiencing metabolic complications and/or the participants were being adequately treated for these risk factors.

A major issue that weight loss programs may face is an increased risk of drop-outs. A study done by Bauer et al. (32) found a drop-out rate of 8% for a 6-month weight loss program, though attrition rates were low this may have attributed to the fact that the cost of the program was fully covered by health insurance in the case of 80% program par-ticipation (7). A study by Ponzo et al. (33) found that a 50% attrition is common among weight loss programs, with rates up to 80% after starting treatment. They found that individuals that dropped out early reported decreased mental well-being. The WLC evaluation saw a dropout rate of 39% which is lower than rates often seen in the literature (53.6%–69.5%) (34-36). Some factors that may have contributed to the lower dropout rate include the multidisciplinary team approach, the active ongoing contact with the participants, and the personalization of the program.

The WLC is a fee for service program. A base fee was charged monthly for participants. Some services offered, including appointments with the RD or psychologist may be covered through individual participants health insurance plans.

### 4.1. Strengths and limitations

A strength of this study is it involves collection of data from an ongoing and existing program, not a program designed to answer a research question. Another strength is the data collection was ongoing prior to the COVID-19 pandemic, creating a unique natural experiment in which looks at the impact of the pandemic. Limitations include the pre-post design, with no control group, and that the impact of COVID-19 included both stoppages in in-person program services due to lockdowns, as well as the heightened stress of living and trying to lose weight during a pandemic. These two factors both could have impaired the WLC program participants success and could not be separated in this study. As well, no exit surveys were collected from participants who dropped out of the evaluation—collecting this data would have provided critical feedback for the program. Finally, the sample size was not based on a power calculation, it was simply dictated by the program capacity, the length of the evaluation period, and the number or program participants who chose to enroll in the evaluation, thus some of the outcomes may have been underpowered.

## 5. Implications for research and practice

Participating in the WLC program prior to the COVID-19 pandemic resulted in weight loss and improvements in waist circumference, systolic blood pressure, diastolic blood pressure, and body fat percentage. This suggests that the program works for many when it can be conducted as originally designed. The COVID-19 pandemic has created additional barriers to weight loss, including but not limited to, inconsistent in-person delivery and its negative impact on participants’ mental health. Long term effects of this pandemic on weight management in adults could exacerbate the problem of obesity in adults, further increasing the prevalence of adults living with obesity (36). The demand for evidence-based weight loss programs post-pandemic will likely be high, and this evaluation suggests that a multi-disciplinary personalized program can be effective. Since most virtual programs are supported by digital platforms, future research should evaluate programs offered virtually that directly address barriers to weight loss created by the COVID-19 pandemic, this could help individuals easily access customized weight loss programs with no interruptions in delivery. As well as, future research should look into types of interventions that evaluate cost effectiveness, to determine if these programs can be covered by healthcare vs. fee based. Since the main focus of this study was weight, measuring dietary and physical changes were challenging, using an actigraphy would be a good way to measure physical activity in future evaluations and measuring dietary change with apps like Keenoa and Rx food may be beneficial (35, 36).

## Data availability statement

The original contributions presented in the study are included in the article/Supplementary material, further inquiries can be directed to the corresponding authors.

## Author contributions

KC collected and analyzed the data. KC wrote the first draft of the paper which was edited by RM, SM, and DM. All authors contributed to the article and approved the submitted version.

## Funding

This evaluation study was funded by the Mitacs Accelerate Fellowship partnered with the Chronic Disease Innovation Centre, which is the research arm of the Wellness Institute and Seven Oaks General Hospital. KC was funded by a Mitacs Accelerate fellowship. Mitacs is a Canadian not-for-profit research and training organization. The funders had a role in the design of the program and in the decision to publish the results. The funders had no role in the collection, analyses or interpretation of the data and in the writing of the manuscript.

## Conflict of interest

The authors declare that the research was conducted in the absence of any commercial or financial relationships that could be construed as a potential conflict of interest.

## Publisher’s note

All claims expressed in this article are solely those of the authors and do not necessarily represent those of their affiliated organizations, or those of the publisher, the editors and the reviewers. Any product that may be evaluated in this article, or claim that may be made by its manufacturer, is not guaranteed or endorsed by the publisher.
