# Supplementary material for: A clinically managed weight loss program evaluation and the impact of COVID-19
Source: Front Nutr. 2023 Jun 16;10:1167813. doi: 10.3389/fnut.2023.1167813 (PMC10311994; doi:10.3389/fnut.2023.1167813)
Supplement: Supplementary file 1 [file Data_Sheet_1.pdf]

Supplementary Material

A Clinically Managed Weight Loss Program Evaluation and the Impact of COVID-19

Katrina Cachero[1*], Rebecca Mollard[2], Semone Myrie[1], and Dylan MacKay[1]

[1]Department of Food and Human Nutritional Sciences, Faculty of Agriculture and Food Sciences, University of Manitoba, Winnipeg, Manitoba, Canada

[2]Chronic Disease Innovation Centre, Seven Oaks General Hospital, Winnipeg, Manitoba, Canada

*** Correspondence:**
Katrina Cachero, kcachero@sbgh.mb.ca
Dylan MacKay, Dylan.Mackay@umanitoba.ca

1 Supplementary Figures and Tables

1.1 Supplementary Figures

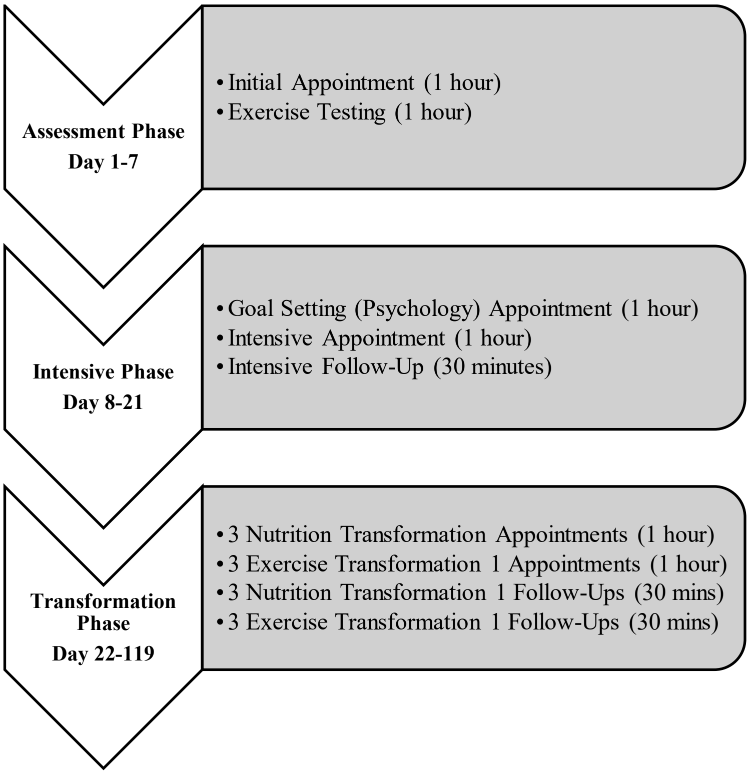

Figure 1. The Wellness Institute Weight Loss Clinic program design. The program evolved in a 3-stage progression. The aim of the first week (Day 1-7) was to get to know the participants, what they were looking to achieve, and assessing their stage of readiness to stage. This included the initial

appointment and exercise testing. The second stage (Day 8-21) is where participants receive a nutrition and exercise prescription based on the interdisciplinary assessment done by the RD and CCPT, as well as a goal setting appointment with the psychologist. The third stage (Day 22-119) includes individual coaching sessions with the RD and CCPT. The exit appointment takes place on Day 120 where outcome measurements are collected.

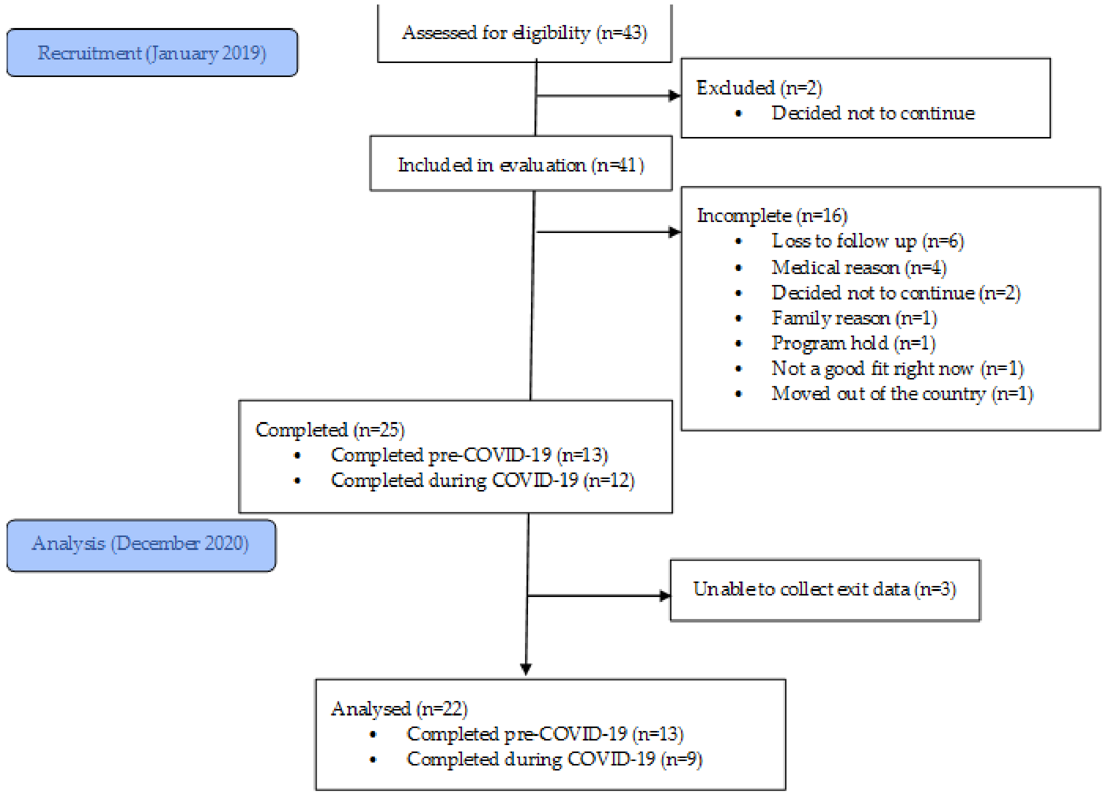

Figure 2. Participant flow diagram from recruitment of participants to analysis of data. A total of 43 participants were enrolled in the evaluation; 16 did not complete, 25 completed and were included at the time of analysis. For those that did not complete the program, reasons for incompletion pre-COVID-19 included loss to follow up (n=5), medical reason unlinked to the program (n=3), family reason (n=1), and decided not to continue (n=1), whereas, for those that completed during COVID-19, reasons for incompletion included loss to follow up (n=2), decided not to continue (n=1) and encountered medical reasons requiring stoppage unlinked to the program (n=1).

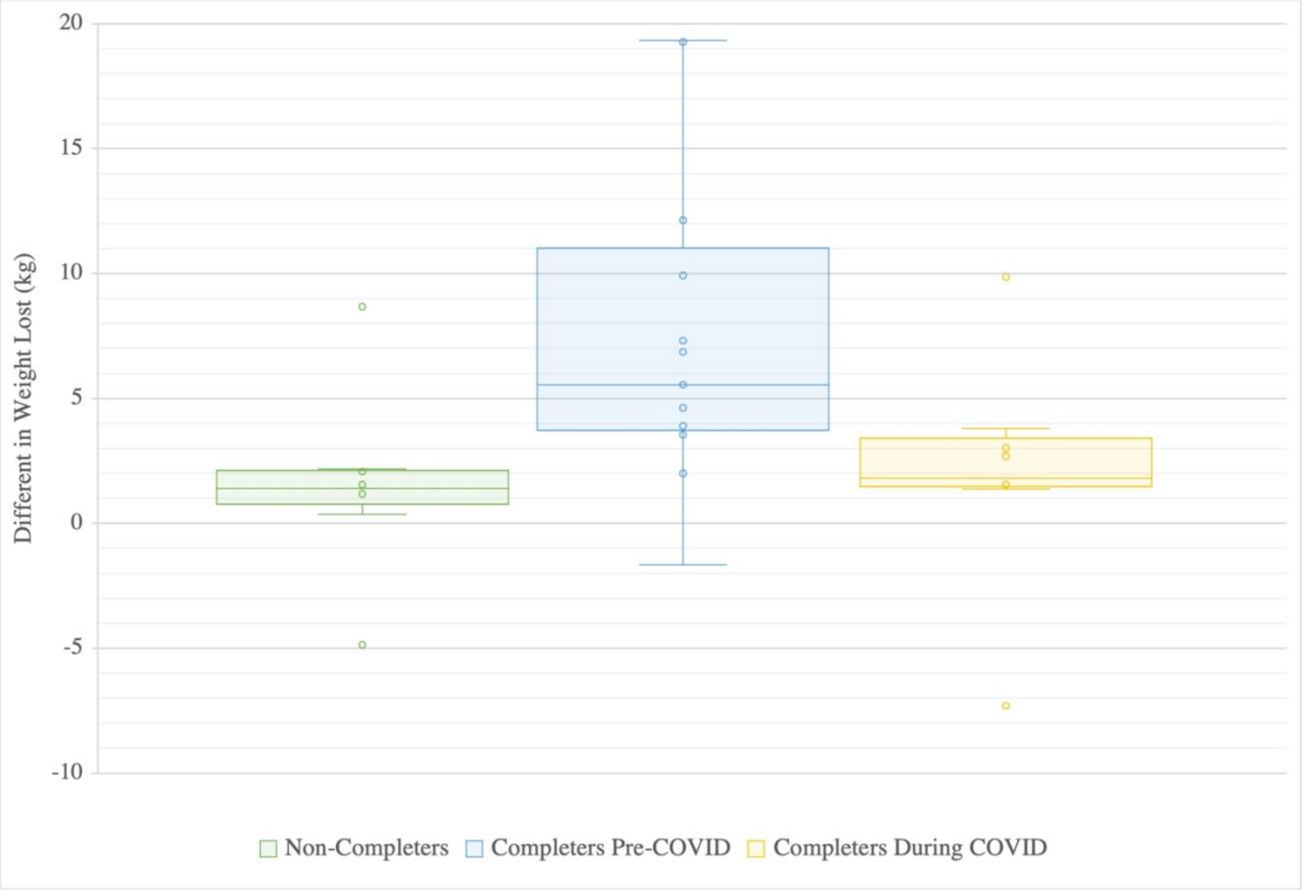

Figure 3. Difference in weight lost for all participants (n=31), categorized by non-completers (n=9), completers pre-COVID (n=13), and completers during COVID (n=9). This shows that completers pre-COVID lost more weight than completers during COVID, where completers during COVID-19 may have gained weight. For non-completers, the last body weight attained by the program was used.

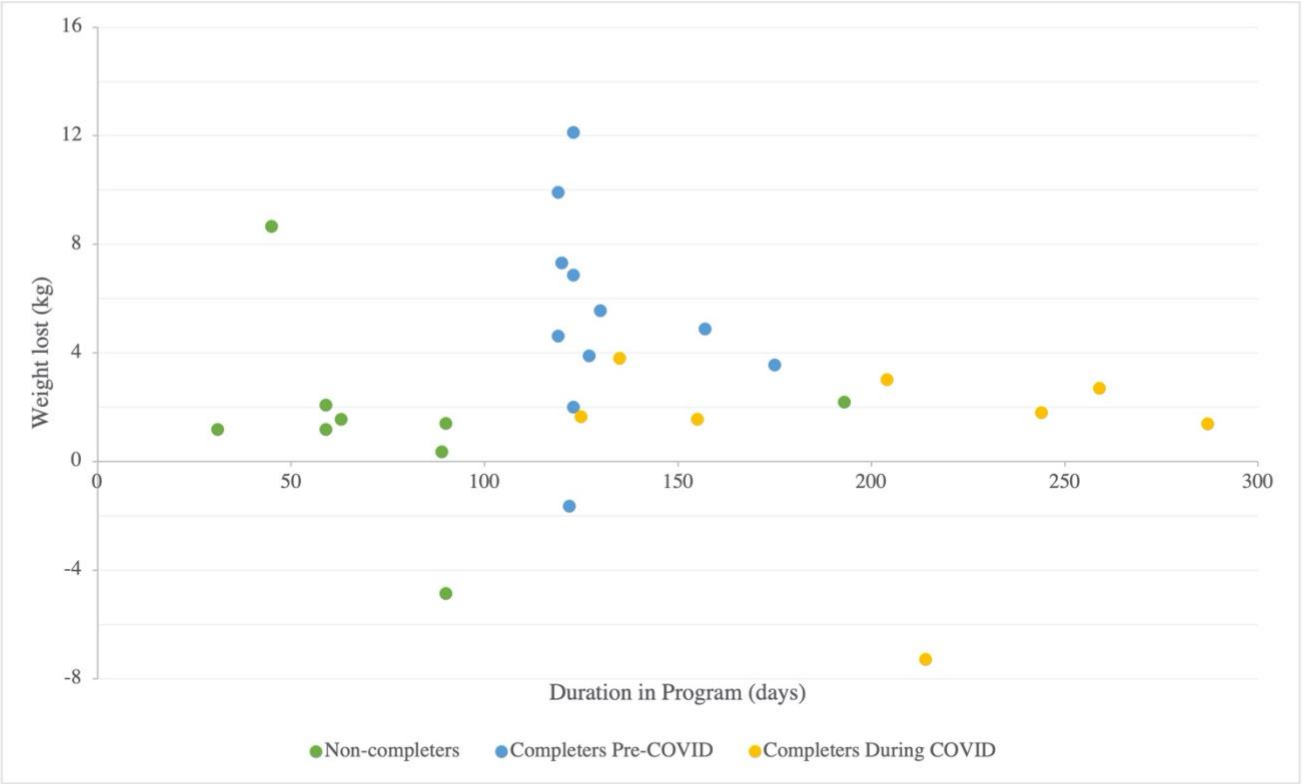

Figure 4. Weight lost versus program duration for all participants (n=31). The impact of time in the program on weight loss is plotted in Figure 3. Participants who completed pre-COVID-19 lost more body weight (-7.51±6.24 kg, $P < 0.001$, n=13) than completers during COVID-19 (-2.05±4.39 kg, $P = 0.19$, n=9).

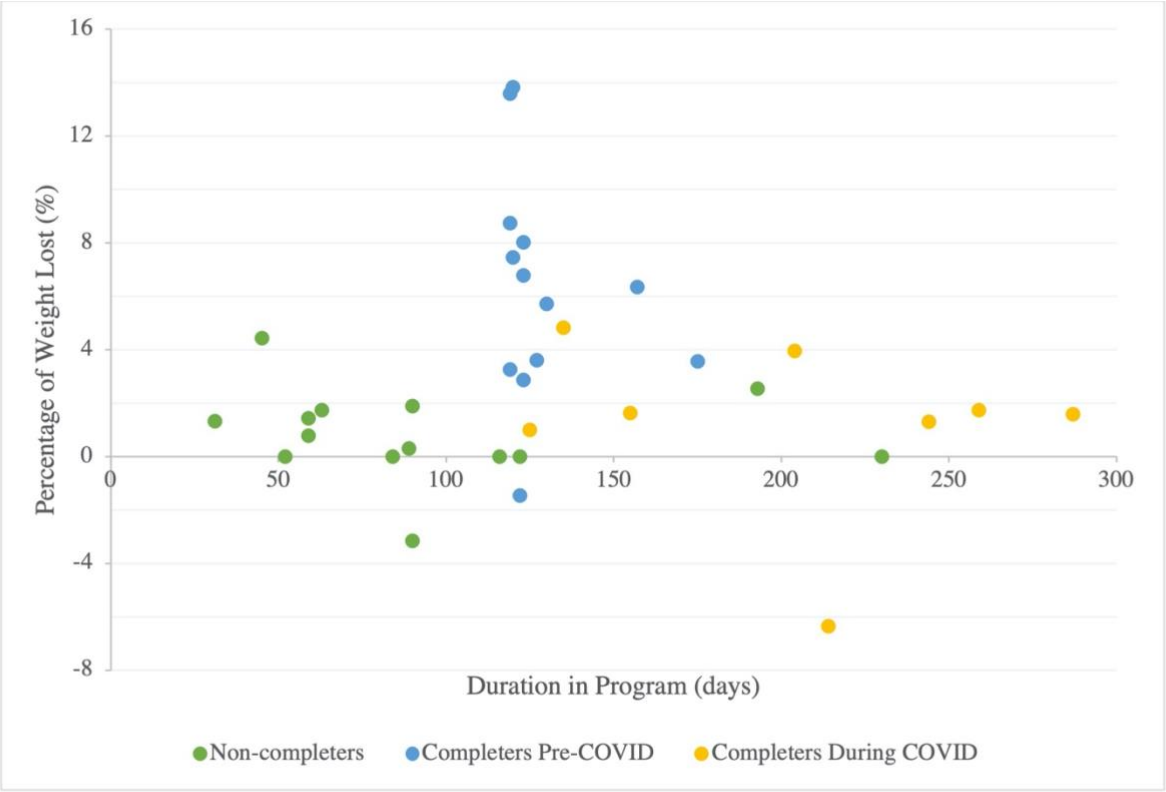

Figure 5. Percentage of weight lost and program duration for all participants (n=31). The impact of program duration on weight loss as a percentage is shown in Figure 4. Completers pre-COVID-19 lost an average of 6.34% body weight, compared to completers during COVID-19 at 2.52% ($P = 0.08$ for pre vs during) and non-completers at 1.50%.
